# The influence of concomitant antidepressant and antipsychotic medication on antidepressant effect and seizure duration of electroconvulsive therapy

**DOI:** 10.3389/fpsyt.2024.1341508

**Published:** 2024-03-18

**Authors:** Sara Mohamad, Samuel Trumm, Sascha Treskatsch, Alisha Drevs, Malek Bajbouj, Leonardo Wiedemann

**Affiliations:** Corporate Member of Freie Universität Berlin and Humboldt-Universität zu Berlin, Department of Psychiatry and Neurosciences, Charité – Universitätsmedizin Berlin, Berlin, Germany

**Keywords:** ECT, electroconvulsive therapy, antidepressants, seizure duration, antipsychotics

## Abstract

**Background:**

A significant proportion of patients with a depressive disorder show resistance to pharmacological and psychotherapeutic antidepressant treatments. Electroconvulsive therapy (ECT) is still one of the most effective treatment methods, especially in the acute phase. In everyday clinical practice, this usually accompanies pharmacological treatment. It has been shown that pharmacological treatment following acute ECT treatment reduces the rate of relapses. However, the effect of various antidepressants (ADs) and antipsychotics (APs) on the effect during the course of ECT has rarely been investigated

**Methods:**

In this retrospective chart review study, the data of 104 depressive patients treated with ECT were examined. We analyzed the influence of concomitant administration of AD and AP or no psychotropic medication on the effect of ECT using the Montgomery–Åsberg Depression Rating Scale (MADRS). We further analyzed the influence of the ADs Bupropion, Venlafaxine, and Sertraline or no AD and the influence of augmentation with Aripiprazole or Quetiapine or Olanzapine.

**Results/discussion:**

Psychotropic medication did not have an impact on antidepressant efficacy of ECT as measured with the MADRS scores. In addition, the comparison between the antidepressant or antipsychotic medications themselves did not show any significant difference. However, we found a significantly different seizure duration depending on the antidepressant substance that patients received during ECT (*p* = .008). ECT treatment itself led to a highly significant reduction of 13.3 points in the MADRS (*p* <.001).

**Conclusion:**

Taken together, our study underlines that concomitant psychotropic medication while doing electroconvulsive therapy does not bare the risk of prolonged seizure duration or does it reduce the effectiveness of ECT. To the best of our knowledge, this study is the first to examine the effect of treatment with antidepressants in combination with antipsychotics while doing ECT. In light of our results, this combination therapy is safe and effective. Bearing in mind the delay in onset of antidepressant action of medication and the importance of antidepressant medication for relapse prevention, this study further supports the recommendation that psychotropic medication should be given in adjunction to ECT.

## Introduction

A major part of patients suffering from depression do not show adequate response to the initial treatment with estimations ranging from 20% to 40% of patients (1). For these patients, having failed first treatment with psychotherapy and antidepressant medication (AD) and after augmentation with further medication, non-pharmacological treatment options exist, with electroconvulsive therapy (ECT) as the treatment of choice. Therefore, most patients who are to receive ECT have already been treated with psychotropic medication. It used to be a clinical practice to discontinue antidepressant agents before ECT due to safety concerns, mainly about prolonged seizures, and the lack of studies examining the effectiveness of a combination therapy (2–4). Nowadays, there is sufficient data on the safety of such a combination, and in more recent guidelines, the combined use of ADs and ECT is allowed, considering this combination to be safe (5). Concerning effectiveness, there is only little evidence. Historical studies from the 1950s to the 1970s examining the use of concomitant TCAs and MAO-inhibitors with ECT showed mixed results (6). A study from 1996 showed a superior effectiveness of the combination of imipramine to ECT compared to paroxetine (7). In a retrospective study by Baghai et al. of 455 patients treated with ECT alone (18.2%) or with ECT in combination with AD, a significant improvement in efficacy was found in the group receiving TCAs, mirtazapine, or SSRIs (8). Consequently, in cases of treatment-resistant depression, the APA recommends a combination therapy to improve the effectiveness of ECT (9). As mentioned above, in severe depression, most patients are treated with antidepressants in combination with non-antidepressants like antipsychotics (APs) before ECT is being evaluated as a further treatment option. Despite this fact, to the best of our knowledge, there is no literature on the effect of a concomitant therapy of antidepressants and ECT vs. a combination of antidepressants and medications used to augment antidepressants and ECT. In this retrospective study, we evaluated the effect of concomitant psychotropic medication (AD vs. AD in combination with AP vs. no medication) in patients with depression on the antidepressant effectiveness of ECT and on seizure duration. We hypothesized that patients receiving psychotropic medication during ECT, whether antidepressants or antidepressants in combination with antipsychotics, do not show a lower response in the antidepressant effect of the treatment and that seizure duration does not change under psychotropic medication.

## Methods

### Montgomery–Asberg depression rating scale

The Montgomery–Åsberg Depression Rating Scale (10) is an external assessment questionnaire to measure symptom severity in depressive disorders. The overall score ranges from 0 to 60 with higher MADRS scores indicating more severe depression. Usual cutoff points are 0–6, normal; 7–19, mild depression; 20–34, moderate depression; 35–60, severe depression (11).

### ECT and data collection

All patients were treated for unipolar or bipolar depression at the Department of Psychiatry and Neurosciences, Charité—Universitätsmedizin Berlin, Germany, and received routine symptom severity testing via the MADRS before the first ECT session and after the acute phase, which refers to the frequency of treatment with three times per week. All patients received unilateral, ultrabrief pulse ECT with a pulse length of 0.25 ms at a frequency of 20 Hz. The intensity is measured as charge in millicoulombs but is often expressed as a percentage representing a patient’s seizure threshold. ECT was performed three times per week and with no set limitation for the number of treatments. At the beginning of each ECT treatment, patients received a sedative and a muscle relaxant intravenously. Only pulses with a pulse length of < 0.5 ms were used with a unilateral electrode placement. Seizure duration was aimed to be between 20 s and 60 s. However, correlations between seizure duration, seizure threshold, and response rate do not seem to exist, as long as a minimum duration can be reached (12). For study purposes, the data of 104 ECT patients treated and tested as mentioned above between 2020 and 2023 were collected. It is important to note that only three of the patients did not fulfill the criteria for treatment-resistant depression. Two of them presented with catatonia and depression, making electroconvulsive therapy (ECT) the first-line treatment, while the third patient declined medication. All other patients, as per the definition of treatment-resistant depression, had completed at least two trials of antidepressants, if not more. It is noteworthy that 27 patients had previously undergone ECT, either within our department or at another facility, which makes 77 in total who received ECT for the first time. Within this cohort, we identified psychiatric comorbidities such as personality disorders (present in seven patients), obsessive–compulsive disorder (OCD, observed in one patient), substance abuse disorders (observed in two patients), panic disorder (two patients) generalized anxiety disorder (three patients), chronic pain disorder (one patient), and post-traumatic stress disorder (PTSD, noted in three patients). We excluded patients with psychotic disorders, defined as patients being diagnosed with a F2x diagnosis by the International Statistical Classification of Diseases and Related Health Problems (ICD), and those lacking baseline and endpoint MADRS scores.

### Data analysis

In this retrospective chart review, patients were divided into three groups depending on their medication: Group A receiving antidepressants alone, Group B receiving antidepressants in combination with antipsychotics, and Group C not receiving any psychotropic medication. From these groups, we then derived in a second explorative analysis substance-specific subgroups: for antidepressants, we evaluated the three medications that were being used most often: Sertraline, Venlafaxine, and Bupropion. For antipsychotics, given as an augmentation to the antidepressant medication, we evaluated Quetiapine, Olanzapine, and Aripiprazole. First, we compared the effect on the difference in MADRS score before and after ECT and on the mean seizure duration between the groups. The pre-treatment MADRS was collected within a week before the first session, and the final MADRS assessments were conducted within a week of the last treatment. Second, antidepressants were compared against each other, as were antipsychotic medications.

Statistical analysis was performed by using SPSS software and included descriptive measures and analysis of variance and Kruskal–Wallis test to compare depressive symptoms and convulsion. A Kruskal–Wallis test was used in cases where a Shapiro–Wilk test showed a non-normal distribution in at least one group, and therefore, an analysis of variance was not feasible. Statistical significance levels were set at *p* <.05.

## Results

In total, data from 104 patients having undergone ECT treatment between the 04/2018 and 06/2023 at Charité—Universitätsmedizin Berlin were included in the analysis. The mean age of patients was 51.54 years at time of treatment. There was a gender distribution of 39 men to 65 women. The number of ECT sessions averaged at 12.9 with a mean seizure duration of 34.8 s and a mean ECT treatment intensity of 49.8%. The aforementioned demographic data are displayed in **Table 1**.

**Table 1:** Demographic and treatment data.

Variables	Total *M* (*SD*)
**Number**	104
**Age**	51.54 (14.32) years
**Sex**	39m, 65f
**Number of** **ECT sessions**	12.9 (2.9)
**Seizure duration**	34.8 (10.6) s
**Treatment intensity**	49.8 (19) %

m, male; f, female; s, seconds; y, years; M, mean; SD, standard deviation.

To test whether the non-randomized groups are comparable, symptoms of depression at the start of treatment and number of ECT sessions were compared between them. To test differences in group means, an analysis of variance was used. No significant differences were found. The results can be seen in **Table 2**.

**Table 2:** Treatment characteristics.

Variables	Total *M* (*SD*)	No medication *M* (*SD*)	Antidepressive medication *M* (*SD*)	Antidepressive & antipsychotic medication *M (SD)*	Mean group difference *p*
**MADRS T0**	32.26 (7.9)	34.52 (7.4)	29.17 (9.3)	32.80 (6.7)	.060
**ECT sessions**	13.35 (2.8)	12.87 (3.1)	13.52 (2.5)	13.54 (3.0)	.642

M, mean; SD, standard deviation; T0, time point of taking MADRS before ECT; p = p-value.

We could not observe any differences between male and female patients regarding MADRS difference or mean seizure duration. These results can be seen in **Table 3**.

**Table 3:** Gender differences.

Variables	Male *M* (*SD*)	Female *M* (*SD*)	Mean group difference *p*
**MADRS T0–T1**	−13.85 (10.79)	12.97 (13.35)	.729
**Mean seizure duration**	33.57 (9.96)	35.62 (11.12)	.390

M, mean; SD, standard deviation; T0, time point of taking MADRS before ECT; T1, time point of taking MADRS after ECT; p = p-value.

There was also no significant interaction between age and difference in MADRS (*p* = .462) or mean seizure duration (*p* = .122).

### Influence of psychotropic medications on antidepressant effect

To test the influence of psychotropic medication on MADRS before and after ECT, we compared the difference in MADRS between the groups receiving no concomitant psychotropic medication to the group receiving antidepressants alone and the group receiving antidepressants and antipsychotics.

In patients with no medication (N = 23), the mean MADRS score decreased by MΔ = −15.22 (SD = 11.12) from MT0 = 34.52 (SD = 7.49) to MT1 = 19.30 (SD = 9.30). In patients treated with antidepressive medication (N = 23), the mean MADRS score decreased by MΔ = −9.52 (SD = 9.99) from MT0 = 29.17 (SD = 9.30) to MT1 = 19.65 (SD = 9.03). In patients treated with antidepressive and antipsychotic medication (N = 35), the mean MADRS score decreased by MΔ = −12.31 (SD = 13.34) from MT0 = 32.80 (SD = 6.71) to MT1 = 20.49 (SD = 14.22).

**Figure 1:**
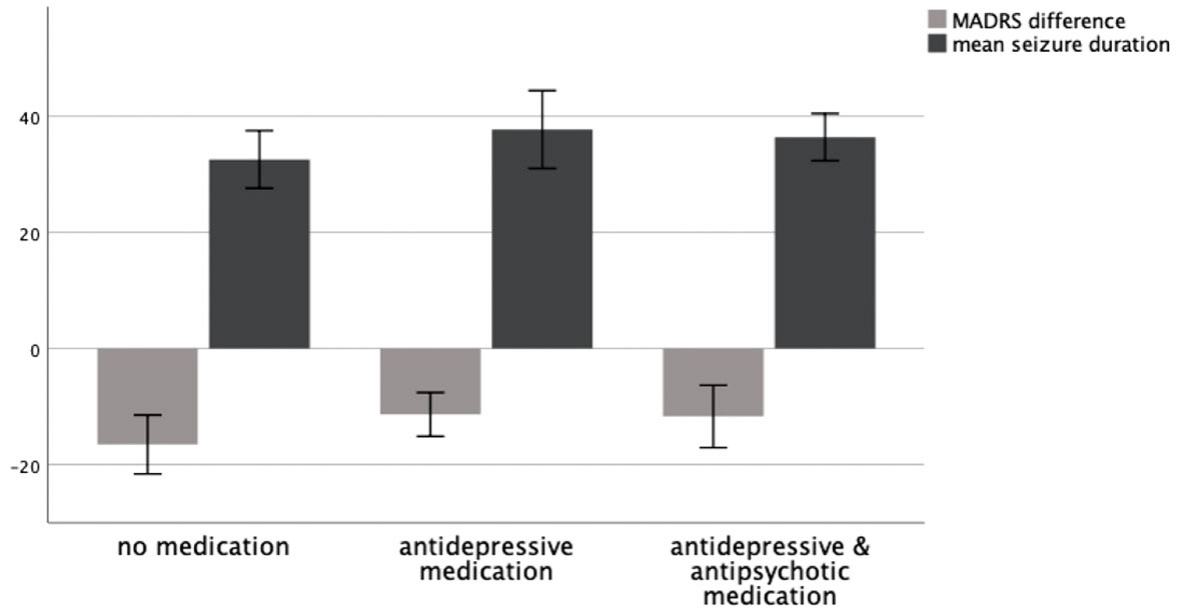
MADRS difference T0–T1 and mean seizure duration in seconds as a function of medication group, 95% CI.

A Kruskal–Wallis test could not confirm the difference in mean MADRS between groups to be statistically significant (*p* = .260). Consequently, patients showed no significantly different change in their depressive symptoms as measured by MADRS depending on whether they received no medication, antidepressive medication, or antidepressive and antipsychotic medication. A graphical representation of group means, and the associated confidence intervals is shown in **Figure 1**.

When considering psychotropic medication given in parallel to ECT, it is mostly prolonged the seizures that are feared, and it has been the rationale to stop psychotropic medication before starting ECT (2). To assess the influence of psychotropic medication on seizure duration during ECT, we compared mean seizure duration between the groups receiving no concomitant psychotropic medication to the group receiving antidepressants alone and the group receiving antidepressants and antipsychotics.

In patients with no medication (N = 17), the mean seizure duration was M = 32.57 (SD = 9.65) seconds. In patients treated with antidepressive medication (N = 17), the mean seizure duration was M = 37.73 (SD = 13.03) seconds. In patients treated with antidepressive and antipsychotic medication (N = 27), the mean seizure duration was M = 36.42 (SD = 10.30) seconds.

A Kruskal–Wallis test could not confirm the difference in mean seizure duration between groups to be statistically significant (*p* = .148). Consequently, patients showed no significantly different seizure duration depending on whether they received no medication, antidepressive medication, or antidepressive and antipsychotic medication. A graphical representation of group means and the associated confidence intervals is shown in **Figure 1**.

### Antidepressant medications

We then further examined the specific antidepressant medications that were being used during ECT treatment in these patients. The mean difference in MADRS before and after treatment was compared between patients being treated with Bupropion, Sertraline, or Venlafaxine.

In patients being treated with Bupropion (N = 9), the mean MADRS score decreased by MΔ = −6.66 (SD = 5.52) from MT0 = 31.89 (SD = 6.80) to MT1 = 25.22 (SD = 6.89). In patients treated with Sertraline (N = 16), the mean MADRS score decreased by MΔ = −16.13 (SD = 10.22) from MT0 = 30.81 (SD = 9.40) to MT1 = 14.69 (SD = 11.45). In patients treated with Venlafaxine (N = 15), the mean MADRS score decreased by MΔ = −13.87 (SD = 13.22) from MT0 = 34.73 (SD = 5.85) to MT1 = 20.87 (SD = 14.34).

**Figure 2:**
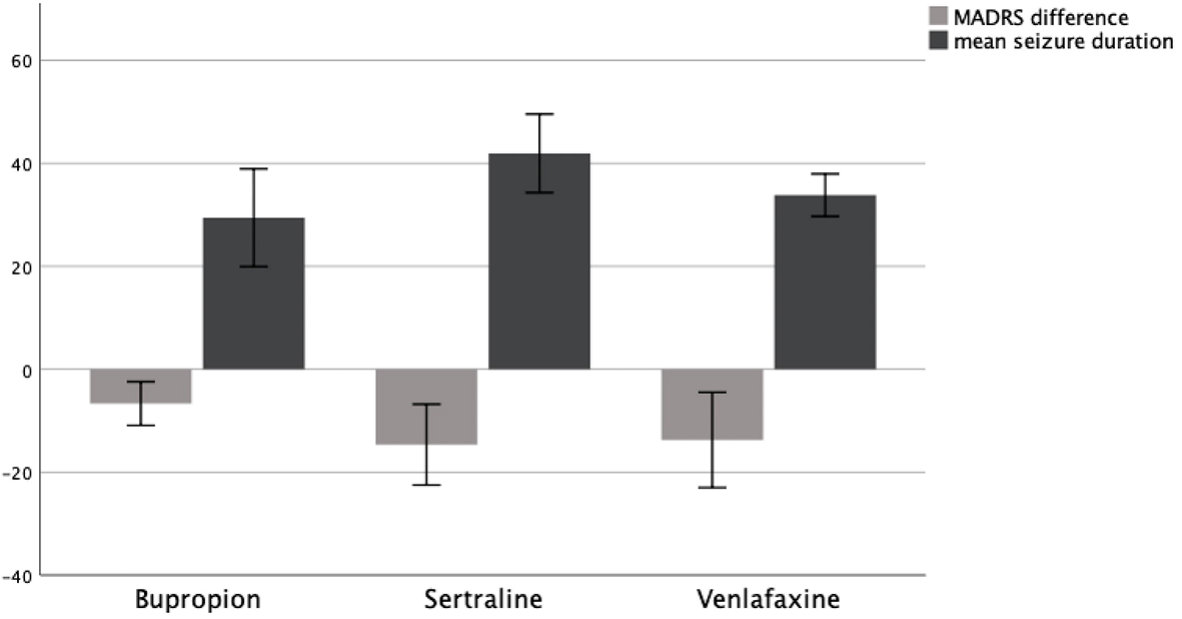
MADRS difference T0–T1 and mean seizure duration in seconds as a function of AD group, 95% CI.

A Kruskal–Wallis test could not confirm the mean difference in MADRS between groups to be statistically significant (*p* = .095). Consequently, patients showed no significantly different change in their depression symptoms as measured by MADRS depending on whether they received Bupropion, Sertraline, or Venlafaxine. A graphical representation of group means and the associated confidence intervals is shown in **Figure 2**.

To assess the influence of different antidepressant medications on seizure duration during ECT, we compared mean seizure duration between patients receiving Bupropion, Sertraline, and Venlafaxine.

In patients receiving Bupropion (N = 9), the mean seizure duration was M = 29.43 (SD = 12.34) seconds. In patients treated with Sertraline (N = 11), the mean seizure duration was M = 41.93 (SD = 11.36) seconds. In patients treated with Venlafaxine (N = 11), the mean seizure duration was M = 33.83 (SD = 6.15) seconds.

A Kruskal–Wallis test confirmed the difference in mean seizure duration between groups to be statistically significant (*p* = .008). Therefore, patients showed a significantly different seizure duration depending on whether they received Bupropion, Sertraline, or Venlafaxine. A graphical representation of group means and the associated confidence intervals is shown in **Figure 2**.

### Antipsychotics

A similar comparison was being done for medications used to augment antidepressants. Here, we evaluated the effect of Aripiprazole, Olanzapine, and Quetiapine, the three medications used most often in our patients.

In patients being treated with Aripiprazole (N = 17), the mean MADRS score decreased by MΔ = −11.35 (SD = 10.62) from MT0 = 30.41 (SD = 8.76) to MT1 = 19.06 (SD = 13.01). In patients treated with Olanzapine (N = 21), the mean MADRS score decreased by MΔ = −14.11 (SD = 19.71) from MT0 = 36.00 (SD = 10.64) to MT1 = 21.89 (SD = 15.57). In patients treated with Quetiapine (N = 9), the mean MADRS score decreased by MΔ = −12.71 (SD = 13.88) from MT0 = 30.57 (SD = 7.30) to MT1 = 17.86 (SD = 14.34).

**Figure 3:**
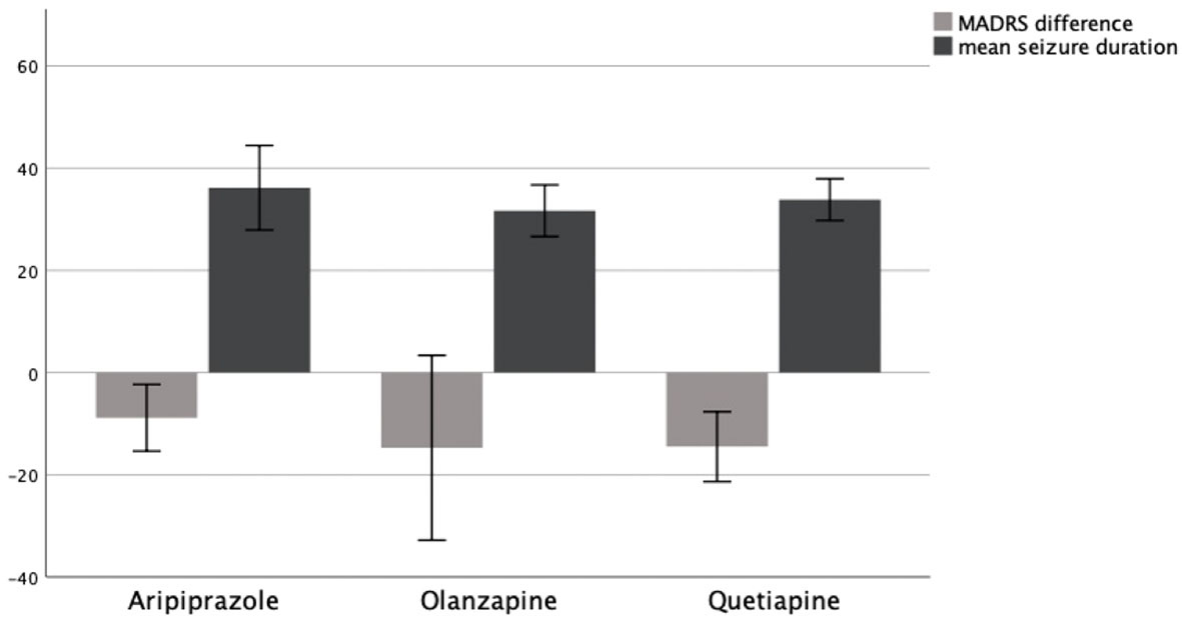
MADRS difference T0–T1 and mean seizure duration in seconds as a function of AP group, 95% CI.

An ANOVA could not confirm the mean difference in MADRS between groups to be statistically significant (*p* = .890). Consequently, patients showed no significantly different change in their depression symptoms as measured by MADRS depending on whether they received Aripiprazole, Olanzapine, or Quetiapine. A graphical representation of group means and the associated confidence intervals is shown in **Figure 3**.

To assess the influence of medications used to augment antidepressants on seizure duration during ECT, we compared mean seizure duration between patients receiving Aripiprazole, Olanzapine, and Quetiapine.

In patients receiving Aripiprazole (N = 13), the mean seizure duration was M = 36.17 (SD = 13.69) seconds. In patients treated with Olanzapine (N = 18), the mean seizure duration was M = 31.67 (SD = 5.45) seconds. In patients treated with Quetiapine (N = 7), the mean seizure duration was M = 33.84 (SD = 8.22) seconds.

An ANOVA could not confirm the difference in mean seizure duration between groups to be statistically significant (*p* = .626). Therefore, patients showed no significantly different seizure duration depending on whether they received Aripiprazole, Olanzapine, or Quetiapine. A graphical representation of group means and the associated confidence intervals is shown in **Figure 3**.

## Discussion

In this retrospective single-center study, we examined 104 psychiatric inpatients diagnosed with depression and treated with ECT regarding the effect of a concomitant psychotropic medication with antidepressants and medications used to augment antidepressants. Our hypothesis was that psychotropic medication does not have a negative impact on the antidepressant effect of ECT or does it change seizure duration.

We found no difference comparing MADRS before and after ECT between patients receiving no psychotropic medication vs. patients receiving antidepressants vs. patients receiving antidepressants and antipsychotics. In addition, when comparing the medications themselves, there was no significant difference, neither in AD nor in AP. In previous studies, the addition of antidepressant medication showed improved results after ECT, which resulted in the APA guidelines stating that antidepressants should be added to ECT for better outcomes (8, 9). Our findings fall in line with many studies showing mixed results, as mentioned by Sackeim et al. (6), and it stands in contrast to findings mentioned ibid with reduced effectiveness of ECT when combined with Venlafaxine. Thus, we could neither confirm an additional antidepressant effect of psychotropic medication to the effect of ECT itself nor a reduced effectiveness by the addition of this medication. Knowing that antidepressant can take several weeks to show an effect and as we do not have data neither on the time that the medication was started nor on MADRS weeks after ECT, we do not know about the course that the different groups would have taken. As we know about the relapse preventing effect after ECT of antidepressant medication (13), it can be assumed that there is a significant difference in MADRS in the course after ECT.

When comparing the different antipsychotic medications, we could not find significant differences in antidepressant outcomes and in seizure duration. This might be due to their relatively low dosage as medications used to augment antidepressants in addition to antidepressants.

With respect to seizure duration, there was a statistically significant difference between the antidepressant medications themselves with longer seizure durations in Sertraline and Venlafaxine compared to Bupropion. Taken together, patients with antidepressants did not show longer seizure durations than patients not receiving concomitant psychotropic medication. Jha et al. (14) describe longer seizure durations under Venlafaxine alone, and Conway et al. (15) show a case report with prolonged ECT seizure under Bupropion in combination with Venlafaxine and Lithium. This study adds to the literature showing that the antidepressants examined, probably generally representing SSRIs, SNRIs, and SNDRIs, can be added to ECT without the risk of prolonged seizure. This seems especially relevant in the face of the fear of many psychiatrists of giving Bupropion during ECT because of case reports of partial status epilepticus under treatment with Bupropion and ECT (16) and because of the warning not to give Bupropion to people with a heightened risk of epilepsy due to its seizure threshold lowering effect (17). The differences could be partially explained by the availability of the extended-release formulation of antidepressants generally and especially of Bupropion. These formulations suggest low occurrence of side effects and especially in the case of Bupropion the seizure threshold lowering effect implicating that the combination of the new formulation should be safe even in combination with antipsychotic and ECT.

Taken together, our study underlines that concomitant psychotropic medication while doing electroconvulsive therapy does not bare the risk of prolonged seizure duration or does it reduce the effectiveness of ECT. To the best of our knowledge, this study is the first to examine the effect of treatment with antidepressants in combination with antipsychotics while doing ECT. In light of our results, this combination therapy is safe and effective. Bearing in mind the delay in the onset of antidepressant action of medication and the importance of antidepressant medication for relapse prevention, this study further supports the recommendation that psychotropic medication should be given in adjunction to ECT. Furthermore, it highlights the safety of a combination of a treatment with ECT and psychotropic medication concerning seizure duration, although we can only tell for the medications examined, being Bupropion, Sertraline, Venlafaxine, Aripiprazole, Olanzapine, and Quetiapine.

The study has several limitations. Due to the statistical tests that were being used, we were not able to state with certainty which of the three groups that were given only antidepressants differed significantly in seizure duration, whether they received Bupropion, Sertraline, or Venlafaxine. But according to the data presented, estimations can be given. Unfortunately, we were not able to make comparisons between different combination therapies of certain antidepressants and antipsychotics patients were treated with, as these groups would have been too small to compare. No follow-up data were available; thus, no assumptions regarding relapse rates can be made. The study did not include comprehensive data on vital parameters, making it challenging to assess the safety of the concurrent medication approach, as there have been reports about potential cardiovascular risks (2, 14). The study also lacks information about the cognitive adverse effects and potential interactions with the anesthetic used during the procedure. This represents an important area for future investigation. As this was a retrospective chart review study, we did not calculate a power analysis *a priori*, as we did not have an influence on the number of patients that were analyzed. However, the lack of a power analysis makes it impossible to state with certainty that the observed lack of interaction between medications and outcome parameters in this study is representative.

## Data availability statement

The raw data supporting the conclusions of this article will be made available by the authors, without undue reservation.

## Ethics statement

Retrospective data analyses for study purposes were approved by the ethics committee of Charité—Universitätsmedizin Berlin. Written informed consent to participate in this study was not required from the participants or the participants’ legal guardians/next of kin in accordance with the national legislation and the institutional requirements.

## Author contributions

SM: Writing – original draft, Writing – review & editing, Conceptualization, Data curation. STru: Writing – original draft, Writing – review & editing, Conceptualization. MB: Supervision, Writing – review & editing. AD: Writing – review & editing, Data curation. STre: Data curation, Formal analysis, Supervision, Writing – review & editing, Resources. LW: Formal analysis, Visualization, Writing – original draft.
